# Accelerated Echo Planer J-resolved Spectroscopic Imaging of Putamen and Thalamus in Obstructive Sleep Apnea

**DOI:** 10.1038/srep31747

**Published:** 2016-09-06

**Authors:** Manoj K. Sarma, Paul M. Macey, Rajakumar Nagarajan, Ravi Aysola, Ronald M. Harper, M. Albert Thomas

**Affiliations:** 1 Department of Radiological Sciences, UCLA Geffen School of Medicine, Los Angeles, CA 90095, USA; 2 UCLA School of Nursing, UCLA Geffen School of Medicine, Los Angeles, CA 90095, USA; 3 Brain Research Institute, UCLA Geffen School of Medicine, Los Angeles, CA 90095, USA; 4 Division of Pulmonary and Critical Care Medicine, UCLA Geffen School of Medicine, Los Angeles, CA 90095, USA; 5 Department of Neurobiology, David Geffen School of Medicine at UCLA, Los Angeles, California, USA.

## Abstract

Obstructive sleep apnea syndrome (OSAS) leads to neurocognitive and autonomic deficits that are partially mediated by thalamic and putamen pathology. We examined the underlying neurochemistry of those structures using compressed sensing-based 4D echo-planar J-resolved spectroscopic imaging (JRESI), and quantified values with prior knowledge fitting. Bilaterally increased thalamic mI/Cr, putamen Glx/Cr, and Glu/Cr, and bilaterally decreased thalamic and putamen tCho/Cr and GABA/Cr occurred in OSAS vs healthy subjects (*p* < 0.05). Increased right thalamic Glx/Cr, Glu/Cr, Gln/Cr, Asc/Cr, and decreased GPC/Cr and decreased left thalamic tNAA/Cr, NAA/Cr were detected. The right putamen showed increased mI/Cr and decreased tCho/Cr, and the left, decreased PE/Cr ratio. ROC curve analyses demonstrated 60–100% sensitivity and specificity for the metabolite ratios in differentiating OSAS vs. controls. Positive correlations were found between: left thalamus mI/Cr and baseline oxygen saturation (SaO_2_); right putamen tCho/Cr and apnea hypopnea index; right putamen GABA/Cr and baseline SaO_2_; left putamen PE/Cr and baseline SaO_2_; and left putamen NAA/Cr and SaO_2_ nadir (all *p* < 0.05). Negative correlations were found between left putamen PE/Cr and SaO_2_ nadir. These findings suggest underlying inflammation or glial activation, with greater alterations accompanying lower oxygen saturation. These metabolite levels may provide biomarkers for future neurochemical interventions by pharmacologic or other means.

Obstructive sleep apnea syndrome (OSAS) is a common sleep disorder, characterized by repeated intermittent hypoxic episodes^1^ from obstructed breathing. The repeated apneas result in multiple cognitive, mood, autonomic, neuroendocrine, and excessive sleepiness deficits, presumably from neural injury in brain structures serving those functions^2^,^3^,^4^,^5^,^6^,^7^. Although multiple brain sites mediate these symptoms, two structures, the thalamus which serves essential sensory processing, cortical activation, and autonomic roles, and the putamen which serves significant autonomic and motor regulatory needs, are of particular importance. Both structures show volumetric and water diffusion changes in OSAS^8^,^9^, but processes underlying the nature of the OSAS injury found in those structures are unclear.

The putamen serves essential roles in autonomic regulation, a major concern in OSAS given the poor sympathetic control in the condition. Damage to the putamen is associated with pathological conditions including Multiple Systems Atrophy^10^. The putamen heavily participates in cognitive behavior, including executive function and working memory, with extensive projections via frontostriatal circuit to the frontal cortex^11^. Injury to the putamen will also affect somatic motor control, which poses concerns for upper airway muscle dysfunction^8^,^12^ underlying airway collapse in OSAS.

The thalamus was selected for evaluation because of its major role in bidirectional transmission of neural signals between cortical and subcortical regions, an interaction significantly affected by sleep states. Specific thalamic nuclei serve sensory focusing roles, and underlie electroencephalographic patterns accompanying attention and redirection of sensory processing. Interruption of thalamic relay circuitry to the cortex, damage to anterior nuclei, or injury to posterior areas interfere with cognitive processing, autonomic, and oxygen and carbon dioxide processing respectively^13^,^14^. The thalamus also interacts with the extended prefrontal neural systems, integrating higher order brain actions with autonomic and inhibitory control functions; dysfunction in those systems could underlie the cognitive deficits found in OSAS^8^.

Both structural and functional neuroimaging studies show brain injury in the putamen^9^,^15^,^16^, and thalamus in OSAS patients^17^,^18^, but information on metabolite levels in damaged areas is sparse^19^,^20^. Magnetic resonance spectroscopy (MRS) is a noninvasive neuroimaging tool that allows assessment of cerebral metabolite changes, providing information on neuronal cellular viability, cellular energetics, and cellular membrane status^20^, and may assist understanding of the nature of the altered thalamic and putamen structures in OSAS.

We examined putamen and thalamic neurochemical changes in OSAS patients using an MRS methodology, accelerated “4D” echo-planar (EP) J-resolved spectroscopic imaging (JRESI), with compressed sensing (CS)^21^, and used prior knowledge fitting (ProFit) algorithms^22^ for metabolite quantification. In the EP-JRESI sequence, an echo planar spectroscopic imaging (EPSI)^23^,^24^ readout was implemented for the acquisition of 2D JRESI^25^ spectra. The EPSI protocol involves a time-varying readout gradient encoding one spatial (*k*_*x*_), and one spectral dimension (*t*_*2*_). JRESI resolves overlapping metabolites better than 1D MRS by taking advantage of J-coupling interactions between protons of metabolites and an extra spectral dimension. Due to the combination of the speed advantage of EPSI readout and increased spectral dispersion offered by 2D JRESI, 4D EP-JRESI enables recording of better-resolved 2D spectra from multiple voxels in a single recording. Despite these advantages, acquisition duration remains a limitation for the routine clinical use of EP-JRESI. A significant acceleration was achieved by using non-uniform undersampling (NUS) along the (*k*_*y*_ *t*_*1*_) plane, and CS^21^,^26^ reconstruction to recover the equivalent missing data to a fully sampled 4D EP-JRESI acquisition. A pilot validation for its application on human brain was performed on a small sample of OSAS subjects^21^.

The purpose here was to determine the processes underlying brain injury in two structures serving critical physiologic and neuropsychologic functions in OSA, using the newly-developed accelerated 4D EP-JRESI methodology. We 1) examined the differences in metabolic profiles between OSAS patients and healthy controls in the putamen and thalamus; 2) determined the sensitivity and specificity of various metabolite ratios for predicting OSAS; and 3) identified the presence and strength of relationships between parameters of sleep disturbance and cerebral metabolic levels. Based on earlier-demonstrated structural and functional alterations in the putamen and thalamus, we hypothesized that altered metabolite integrity would be present, and help reveal the nature of previously-shown injury.

## Materials and Methods

We assessed fifteen OSAS patients (age 50.3 ± 11.6 years; apnea-hypopnea index (AHI) 29.55 ± 15.6 events/hour; 2 mild, 6 moderate and 7 severe), and twenty six age-matched healthy controls (HC) (49.1 ± 10.2 years). OSAS patients were recruited following a sleep study at the University of California at Los Angeles (UCLA) Sleep Disorders Center, and diagnosed on criteria of overnight polysomnography (PSG) scores according to the 1999 American Academy of Sleep Medicine guidelines^27^. Two patients were diagnosed within the prior month, three subjects within 2 years, and the remainder had experienced up to 5 years of the disorder. AHI, baseline, and minimum oxygen saturation values were determined. The AHI index is the average number of disordered breathing events, which comprises both apneas and hypopneas, per hour, and OSAS is defined as an AHI of 5 or greater with associated daytime symptoms. Evidence of clinical pathological findings or additional illnesses, including a previous history of heart failure, stroke, diagnosed cerebral conditions, psychiatric disease, trauma or metallic implants, and current history of cardiovascular-altering medications or any mood-changing drugs was cause for exclusion for both OSAS and HC. HC were healthy individuals with no sleep disorder, based on interviews and screening questions. The study was approved by the Institutional Review Board at UCLA, and all methods were carried out in accordance with the approved guidelines. Written informed consent was obtained from all individuals before participation.

All data were collected on a 3T Trio-Tim MRI scanner (Siemens Medical Solutions, Erlangen, Germany) using an 8-channel phased-array head coil. All participants were instructed to limit head motion and remain still during scanning. Additionally, once the participant was positioned supine on the scanner gantry with the head in a midline location in the coil, foam padding was used to further minimize head motion. Before performing the NUS-based 4D EP-JRESI^21^ sequence, 3D high resolution T_1_-weighted images were acquired using a Magnetization Prepared Rapid Gradient Echo (MPRAGE) pulse sequence (TR/TE = 2200/2.34 ms; inversion time = 900 ms; flip angle = 9°; matrix size = 320 × 320; FOV = 230 mm × 230 mm; slice thickness = 0.9 mm; number of slices = 192) in the sagittal plan for voxel localization. The 4D EP-JRESI sequence (90^°^-180°-*t*_*1*_-180°) was modified, imposing NUS along *k*_*y*_*t*_*1*_ plane^21^ to sample only 25% of the fully sampled signal. NUS-based EP-JRESI was performed over a coronal slice covering the putamen and thalamus using the following parameters: TR/TE = 1.5 s/30 ms, FOV = 24 × 24 cm^2^ with 16 × 16 grids, slice thickness = 1.5 cm, voxel resolution = 3.37 cm^3^, 512 bipolar echo pair, 64∆t_1_ increments (1 ms), averages = 2, and *F*_1_ and *F*_2_ bandwidths of 1000 Hz and 1190 Hz, respectively for a scan time of approximately 12 minutes. In post-processing, the data first had a frequency-dependent linear phase correction applied to provide a maximum echo sampling scheme^28^, resulting in a bandwidth of ±250 Hz along F_1_. Outer volume saturation bands were included outside the PRESS volume of interest. Voxel shim and water suppression were manually adjusted, and a line width of ~12–14 Hz was achieved. This process was followed by a non-water-suppressed scan fully sampling *k*_*y*_ with only the first *t*_1_ increment to be used for eddy current correction and as a reference for coil combination, adding 30 seconds to the total scanning duration.

The acquired undersampled data were reconstructed using a modified Split Bregman algorithm^29^. The reconstruction was performed over each coil separately, and the individual coil reconstructions were combined as a sum-of-squares. Reconstructed data were further post-processed using a series of steps described elsewhere^21^, with a custom MATLAB-based program.

Metabolite ratios with respect to the creatine (Cr; 3.0 ppm) (S/S_Cr_) peak were calculated using the Profit algorithm^22^, optimized for processing the Siemens data, based on a linear combination of 2D model spectra. ProFit performs a hybrid time and frequency domain fitting using a non-linear outer loop and an inner linear least-square fit for the concentrations incorporating the maximum available prior knowledge. Before starting the fitting procedure, extracted 2D J-resolved spectra were subjected to zeroth-order phase correction and frequency shifts along F_1_ and F_2_ dimensions. The GAMMA (general approach to magnetic resonance mathematical analysis)^30^ library was used to simulate the prior knowledge basis set using chemical shifts and J-coupling values reported in the literature^31^, and was exposed to the same post-processed steps as the actual *in-vivo* data. The basis set contained a set of 19 basis metabolites: Cr, N-acetylaspartate (NAA), phosphorylcholine (PCh), free choline (Cho), glycerylphosphocholine (GPC), γ-aminobutyric acid (GABA), glutamine (Gln), glutamate (Glu), glutathione (GSH), myo-inositol (mI), N-acetylaspartylglutamate (NAAG), phosphoethanolamine (PE), scyllo-Inositol (Scy), taurine (Tau), lactate (Lac), aspartate (Asp), glucose (Glc), glycine (Gly), ascorbic acid (Asc), threonine (Thr), and alanine (Ala). The quality of the fit was individually evaluated for each metabolite using the Cramer-Rao Lower Bound (CRLB)^32^ values. In the present study, we report metabolite ratios with respect to Cr; existing studies do not show Cr changes in OSAS subjects. No attempts were made for absolute quantification, although they can be determined from the reported concentrations of Cr.

### Statistical analysis

All statistical analyses were performed using SPSS software (version 23.0, IBM Corporation, Armonk, NY). The metabolite differences between OSAS patients and healthy controls were tested with analysis of covariance (ANCOVA), with age and gender as covariates. Receiver operating characteristics (ROC) curve analyses based on logistic regression models were performed to identify the optimal cutoff value for the metabolites that show significant differences between the two groups. The area under the curve (AUC), interpreted as the average value of sensitivity for all possible values of specificity, was taken as a criterion for the success of the ROC analysis. Sensitivity, specificity, and accuracy were reported for the optimal thresholds calculated based on the Youden index^33^. Pearson’s correlations were performed in OSAS patients to explore relationships between metabolite ratios and sleep parameters (AHI, oxygen saturation variables). The level of statistical significance was set at *p* < 0.05 for all analyses.

Reproducibility and reliability of EP-JRESI were assessed by finding the co-efficient of variance (COV) for three data sets in different day sessions each for a healthy control and OSAS subject.

## Results

**Figure 1:**
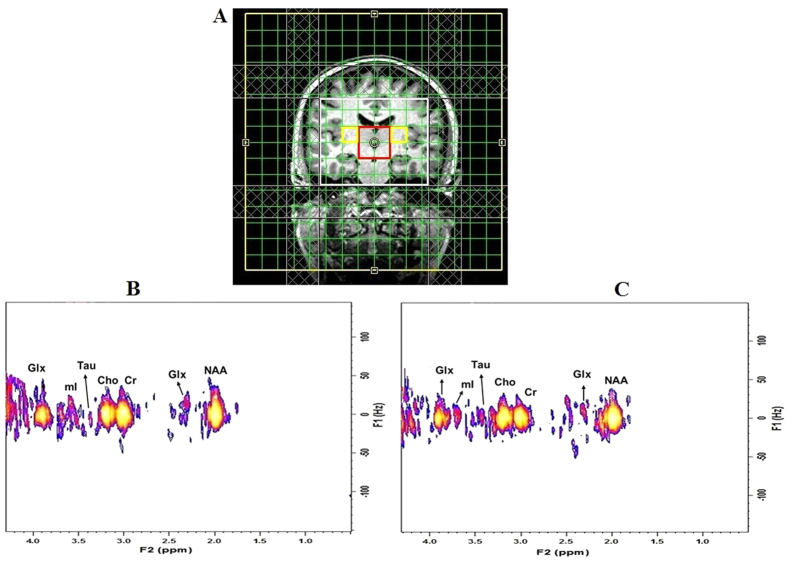
(**A**) Voxels representing the putamen (yellow) and thalamus (red) overlaid on the T1-weighted localization image of a 59-year old OSAS patient; selected 2D J-resolved spectra extracted from (**B**) right putamen and (**C**) right thalamus.

Demographic and sleep variables of OSAS patients and healthy controls are summarized in Table 1. No significant differences appeared between groups in age (p = 0.84), or gender (p = 0.44). Figure 1(A) shows voxel locations on a T_1_-weighted axial brain MRI of a 59-year-old OSAS patient. A representative 2D J-resolved spectrum, extracted from the right thalamus and putamen region of the same subject and then CS reconstructed, is shown in Fig. 1(B,C), respectively.

Metabolite ratios with respect to Cr in the right and left thalamus of OSAS patients and healthy controls groups are shown in Table 2. We observed increased mI/Cr ratios bilaterally in the thalamus of OSAS patients over healthy controls. Increased Glx/Cr and Glu/Cr were found in right thalamus, and decreased tNAA/Cr and NAA/Cr in the left thalamus, respectively. The thalamus showed significantly reduced tCho/Cr bilaterally. We also found significantly decreased GPC/Cr, and increased Gln/Cr and Asc/Cr in the right thalamus of OSAS patients.

**Figure 2:**
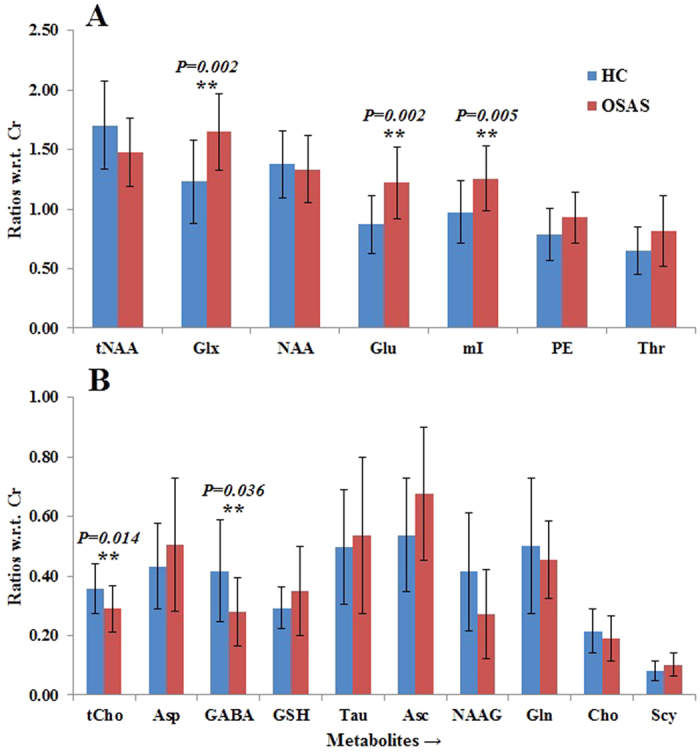
Bar graphs showing mean metabolite ratios ( ±SD) with respect to Cr in the right putamen. **Significant at the 0.05 level.

**Figure 3:**
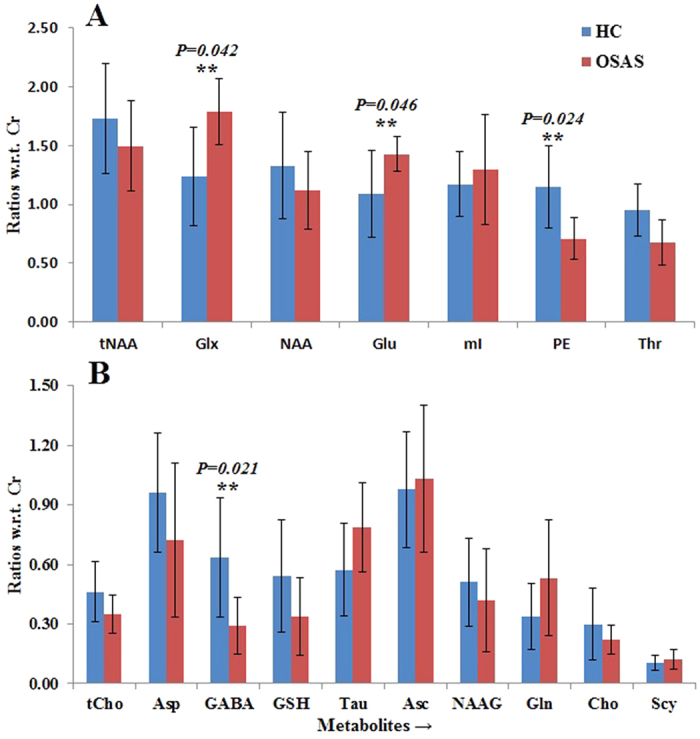
Bar graphs showing mean metabolite ratios (±SD) with respect to Cr in the left putamen. **Significant at the 0.05 level.

Figures 2 and 3 show the metabolite ratios in the right and left putamen of OSAS patients and healthy controls, respectively. Increased Glx/Cr and Glu/Cr, and reduced GABA/Cr appeared in the bilateral putamen of OSAS patients, compared to healthy controls. In the right putamen, we found increased mI/Cr ratios and decreased tCho/Cr ratios. The left putamen exhibited decreased PE/Cr ratios. In addition, as in the thalamus, a similar trend of decreased tNAA/Cr and NAA/Cr appeared in the bilateral putamen. Apart from the patterns described above, no other metabolite ratios displayed a significant difference between the healthy control and OSAS groups.

**Figure 4:**
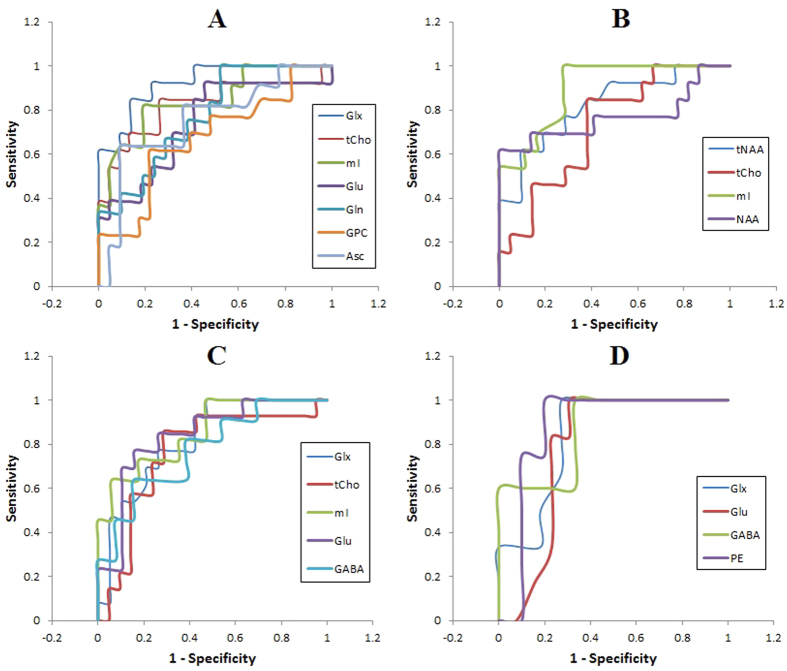
Receiver operating characteristic (ROC) curve for the cutoff value of the metabolite ratios showing significant differences between OSAS patients and healthy controls in the (**A**) right thalamus, (**B**) left thalamus, (**C**) right putamen, and (**D**) left putamen.

Detailed results of the ROC curve analyses in Table 3 and Fig. 4 provide the sensitivity, specificity, accuracy, and AUC for classifying the OSAS patients versus healthy controls in bilateral putamen and thalamus. In the right thalamus, the Glx/Cr ratio showed the highest AUC (0.92) with sensitivity, specificity, and accuracy of 84.6%, 86.4% and 85.7% respectively, at a cutoff value of 1.5, compared to other metabolites. Among the left thalamus metabolite ratios, mI/Cr gave the highest AUC and ROC values for differentiating OSAS patients and healthy controls, with an optimal cutoff value of 1.1. For the right putamen, Glu/Cr and PE/Cr ratios had the highest AUC respectively. In the right putamen, the optimal off value for Glu/Cr was 1.1, indicating a sensitivity of 76.9% and specificity of 84.2%. The PE/Cr ratio in the left putamen showed a 100% sensitivity and 80% specificity, with a threshold cut-off value of 0.95 in the ROC curve for the diagnosis of OSAS.

**Figure 5:**
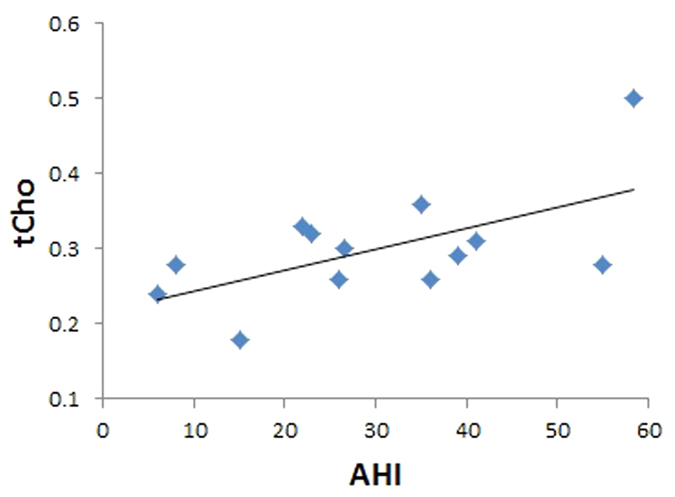
Relationship of tCho/Cr ratio with AHI in OSAS patients.

Table 4 and Fig. 5 show significant correlations between sleep variables and metabolite ratios in the OSAS patient group. In the left thalamus, positive correlations appeared between the mI/Cr ratio and SaO_2_ baseline. Positive correlations also emerged between AHI and right putamen tCho/Cr ratio, SaO_2_ baseline, and right putamen GABA/Cr ratio and SaO_2_ baseline, and left putamen PE/Cr ratio and SaO_2_ nadir and left putamen NAA/Cr ratio. A negative correlation was found between SaO_2_ nadir and left putamen PE/Cr ratio.

Table 5 demonstrates the reproducibility (n = 3) results. The coefficients of variation (CV) of NAA, Cho, mI, tNAA, tCho and Glx ratios both in HC and OSAS were under 20%.

## Discussion

OSAS patients exhibit neurochemical alterations and changes in metabolite levels in the thalamus and putamen. A number of processes, possibly including intermittent cerebral ischemia, marked blood pressure swings, and CO_2_ changes accompanying repeated apnea episodes during sleep in OSAS patients cause structural injury and dysfunction^3^,^6^,^7^. Although atrophy and neuronal loss in the thalamus and putamen of OSAS patients have been reported earlier^15^,^16^,^17^,^18^, the underlying pathophysiology underlying the injury is complex, with the putamen also showing regions of volume increase suggestive of inflammation or glial activation, as opposed to cell death or reduced myelin integrity. We found significantly lower tCho/Cr ratios in the bilateral thalamus and right putamen, tNAA/Cr and NAA/Cr in left thalamus, GABA/Cr in bilateral putamen, PE/Cr in left putamen and GPC/Cr in the right thalamus in the OSAS patients, as compared to healthy controls. We also found increased Glx/Cr and Glu/Cr in bilateral putamen and right thalamus, mI/Cr in bilateral thalamus and right putamen, Gln/Cr and Asc/Cr in the right thalamus in OSAS subjects over control subjects. The neurochemical findings suggest that the pathology in the thalamus and putamen in OSA is not just tissue loss due to neuronal death, but that other, additional tissue changes occur. We speculate that those changes are of an inflammatory nature, or related to glial changes^34^,^35^,^36^.

The thalamus has the potential to substantially impact the characteristics of OSAS. The structure consists of multiple nuclei, serving roles in sensory selection as well as significant O_2_ and CO_2_ regulation^37^,^38^,^39^. Thalamo-cortical interactions underlie electroencephalographic characteristics of waking and sleep^40^. Both the putamen and thalamus have reciprocal projections to limbic and cortical structures that regulate neuropsychological and autonomic functions affected in OSAS^13^,^41^. Such projections would alter information transfer between multiple brain sites, leading to impaired functions consistent with multiple symptoms of OSAS. The nature of the neurochemical changes is more consistent with functional change and reorganization, rather than solely reduced-function (i.e., less activation) and cell death.

Four-dimensional EP-JRESI retains many of the benefits of 1D MRS, but disperses the overlapping resonances into a second dimension, reducing congestion, and increasing metabolite specificity. Compared to regular 2D MRS, 4D EP-JRESI also enables recording 2D spectra from multiple voxels in a single scan. The present data include measures of metabolites earlier detected with conventional, 1D MRS, specifically Cho, Cr, mI and NAA. The current procedures could detect metabolites not quantifiable with 1D MRS, and additionally include GABA, GSH, and Asc. Our findings of reduced tNAA/Cr, NAA/Cr, and increased mI/Cr are consistent with findings in 1D MRS studies performed in frontal, parietal, and occipital cortices, as well as in thalamus and other regions^20^,^42^,^43^,^44^,^45^,^46^,^47^.

tNAA (NAA), considered a marker of neuronal viability, is predominantly located in neurons, and biosynthesis occurs at both microsomal and mitochondrial sites. Reduction of tNAA/Cr, and NAA/Cr in the left thalamus of OSAS patients in our study could reflect neurodegeneration and chronic neural injury. The bilateral putamen and right thalamus revealed no significant changes in tNAA/Cr and NAA/Cr ratios from those of the control group, suggesting no, or little impact of OSAS. The reason for minimal alterations may be the absence of an accumulated neuronal loss in some patients who were newly diagnosed, or the dominance of structural changes other than cell death (inflammation, gliosis). mI is preferentially concentrated in glial cells, and is involved in signal transduction pathways^41^; thus, increased mI/Cr ratios here may reflect increased glial activation^41^ and reactive gliosis^42^ in those areas, which could result in increased inflammatory action leading to more neuronal injury in a cumulative process from ongoing repeated episodes of hypoxia in OSAS patients.

Decreased tCho/Cr ratios in bilateral thalamus and right putamen, as well as reduced GPC/Cr ratios in right thalamus of OSAS patients over healthy controls are consistent with previously-reported results of Cho/Cr reduction in the frontal lobe by Alchanatis *et al*.^42^. Cho peaks including GPC, PCh and free Cho represent cell membrane density and turnover. Reduced tCho and GPC ratios may indicate loss of myelin lipids or dysfunction of phospholipid metabolism^42^. As suggested by Alchanatis *et al*.^42^, a possible interpretation for the lower Cho is that OSA induces brain metabolic impairment through a combination of haemodynamic impairment, sleep fragmentation, and intermittent hypoxia, leading to decreased membrane turnover and possible apoptosis.

GABA is a major inhibitory neurotransmitter in the adult brain, with level alterations, such as reduced GABA/Cr ratios in the bilateral putamen here potentially having serious functional consequences. Reduced GABA levels appear in insular cortex of OSAS^48^, other sleep disturbances, including primary insomnia^49^,^50^, major depressive disorder, and anxiety disorders^51^; OSAS is a major risk factor for the latter two disorders. It should be noted that the GABA/Cr ratios were slightly overestimated, possibly resulting from using a TR of 1.5 s, a time during which metabolites are not fully recovered. In addition, we used no T_1_ or T_2_ correction. However, the ProFit results were very consistent and reproducible. Phosphoethanolamine (PE) shows a strong structural similarity to GABA. PE is a precursor of phospholipid synthesis, which is decreased in Alzheimer’s disease brain^52^. The finding of decreased PE/Cr ratios in the bilateral putamen of OSAS here may indicate phospholipid breakdown.

High Glu could reflect both a functional reorganization, and, at high levels, the potential for the neurodegenerative process of excitotoxicity. Glu is a powerful excitatory neurotransmitter, while Gln is the most prevalent glutamate precursor in synaptic terminals. Normally, Glu plays an important role in learning and memory^53^, with abnormally high Glu levels leading to over-excitation of the receiving nerve cell, causing cell damage and/or death. The high Glu/Cr, Glx/Cr, Gln/Cr found here may reflect damaging excitotoxic processes arising from intermittent hypoxia^34^, a process that can occur extremely rapidly^54^. However, the high glutamate measured here was in whole tissue, and does not necessarily correspond to concentrations in extracellular fluid, where the neurochemical is key for excitotoxicity. It has also been reported that increased Gln levels stimulate the brain, preventing deep sleep^55^. Fatty acids are produced under hypoxia, primarily synthesized from glutamine carbon via the reductive pathway. The finding of increased Asc in the right thalamus could result from the oxidative stress occurring in OSAS^56^.

The ROC curve analyses demonstrate high sensitivity and specificity for the metabolite ratios in differentiating OSAS patients from healthy controls. Glx/Cr was the best predictor in right thalamus, and mI/Cr in left thalamus. In right and left thalamus, Glu/Cr and PE/Cr had the best sensitivity and specificity, respectively. The findings suggest metabolite ratios might be an important indicator of hypoxic cerebral impairment in OSA, with combinations of metabolite ratios allowing for higher sensitivity and specificity.

The test-retest results demonstrated metabolite ratios were reproducible, as reflected in small COV values over three different time-points. The COV values were within the observed standard deviation (SD) range for the respective metabolites, both in healthy controls and OSAS subjects.

The limitations of this study would contribute towards false negatives rather than false positives; thus, the findings of differences in OSAS are unlikely to be over-estimated. One limitation is that OSAS patients were not categorized according to disease severity. The AHI values showed a patient pool with mixed numbers of mild, moderate, and severe OSAS subjects; mild levels of OSA are likely much less injurious than severe cases. Secondly, the basis set for ProFit processing included prior-knowledge spectra of metabolites only; thus, addition of prior-knowledge for macromolecules and lipids may improve quantitation accuracy. Thirdly, the findings are limited in specificity, because, although we excluded subjects with common ischemic and metabolic conditions, that exclusion was only verbally ascertained, and some patients with other conditions may have been included. A fourth limitation was the small number of OSAS patients. Higher numbers of patients would allow categorization of subjects into mild, moderate, and severe OSAS, and separate analyses could be performed between the more homogenous groups and control subjects, enhancing ROC accuracy.

## Conclusions

Patients with OSAS showed altered neurochemical levels in the thalamus and putamen suggestive of substantially elevated glial responses to hypoxia and other stresses, and the presence of mild neurodegenerative processes. These results show cerebral metabolite changes associated with OSAS in the thalamus and putamen, areas with functions related to symptoms of this disorder, and which have been affected structurally in other neuroimaging studies. Our findings using the accelerated 4D EP-JRESI method are in broad agreement with the outcomes previously demonstrated using 1D MRS, are consistent with the known phenomena associated with oxidative stress in OSAS, and expand the number of affected metabolites. The findings help explain the processes underlying the structural brain changes found in thalamic and putamen regions in OSAS, and raise the possibility of targeting neurochemicals to intervene in the syndrome. Most of these metabolites can be manipulated through pharmacological approaches; the MRS technique used here could serve as a biomarker for any such intervention.

## Additional Information

**How to cite this article**: Sarma, M. K. *et al*. Accelerated Echo Planer J-resolved Spectroscopic Imaging of Putamen and Thalamus in Obstructive Sleep Apnea. *Sci. Rep.* **6**, 31747; doi: 10.1038/srep31747 (2016).

## Figures and Tables

**Table 1:** Demographic and sleep variables for OSAS patients and healthy controls.

**Parameters**	**OSAS (Mean** ± **SD)**	**Healthy Controls (Mean** ± **SD)**	***p*****-values**
Age (Years)	50.27 ± 11.6	49.12 ± 10.1	0.84
Sex (Male:Female)	(10:5)	(16:12)	0.44
BMI (kg/m^2^)	31.15 ± 4.6	26.51 ± 6.8	0.06
AHI	29.55 ± 15.6	—	—
SaO_2_ Baseline	94.20 ± 2.6	—	—
SaO_2_ Nadir	82.46 ± 8.2	—	—

**Table 2:** Comparison of selected ProFit-quantified metabolite ratios (Mean ± SD) in the right and left thalamus of HC and OSAS patients.

**S/S**_**Cr**_	**Right Thalamus**			**Left Thalamus**		
	**OSAS**	**HC**	***P*****-values**	**OSAS**	**HC**	***p*****-values**
tNAA	1.44 ± 0.26	1.53 ± 0.24	0.247	1.27 ± 0.25	1.59 ± 0.26	0.002**
Glx	1.79 ± 0.36	1.28 ± 0.21	0.000**	1.75 ± 0.44	1.57 ± 0.49	0.488
tCho	0.32 ± 0.08	0.42 ± 0.13	0.018**	0.29 ± 0.08	0.37 ± 0.10	0.012**
NAA	1.21 ± 0.17	1.29 ± 0.31	0.479	0.98 ± 0.26	1.21 ± 0.21	0.017**
Glu	1.20 ± 0.39	0.94 ± 0.19	0.009**	1.27 ± 0.48	1.26 ± 0.58	0.678
mI	1.39 ± 0.57	0.87 ± 0.25	0.001**	1.49 ± 0.28	0.95 ± 0.29	0.000**
GABA	0.35 ± 0.12	0.41 ± 0.16	0.213	0.47 ± 0.20	0.51 ± 0.25	0.505
GSH	0.40 ± 0.10	0.37 ± 0.16	0.654	0.41 ± 0.14	0.53 ± 0.24	0.132
PE	0.66 ± 0.29	0.77 ± 0.28	0.370	0.85 ± 0.32	0.86 ± 0.25	0.612
Tau	0.70 ± 0.28	0.62 ± 0.29	0.572	0.57 ± 0.25	0.61 ± 0.18	0.713
Asc	0.77 ± 0.14	0.63 ± 0.17	0.046**	0.85 ± 0.30	0.98 ± 0.46	0.409
GPC	0.22 ± 0.07	0.28 ± 0.10	0.037**	0.21 ± 0.05	0.26 ± 0.11	0.053
Cho	0.12 ± 0.05	0.13 ± 0.06	0.681	0.10 ± 0.05	0.11 ± 0.05	0.605
Scy	0.11 ± 0.05	0.10 ± 0.02	0.383	0.11 ± 0.06	0.10 ± 0.04	0.500
Thr	0.94 ± 0.26	0.86 ± 0.28	0.540	0.95 ± 0.23	0.91 ± 0.33	0.988
Gln	0.65 ± 0.32	0.41 ± 0.15	0.017**	0.52 ± 0.22	0.46 ± 0.20	0.668
Asp	0.64 ± 0.21	0.62 ± 0.29	0.902	1.02 ± 0.35	1.02 ± 0.44	0.948

^**^tCho = *Cho* + *GPC* + *PCh, tNAA* = *NAA* + *NAAG, Glx* = *Glu* + *Gln.*

**Table 3:** Measures of sensitivity, specificity, accuracy, AUC, and Cut Off values using significantly different metabolite ratios in the four ROI in discrimination of OSAS patients from healthy controls with receiver operating characteristic curve analysis.

	**Cut Off**	**Sensitivity %**	**Specificity %**	**Accuracy %**	**AUC %**
Right Thalamus					
Glx	1.5	84.6	86.4	85.7	92.3
tCho	0.4	84.6	73.9	77.8	82.3
mI	1.1	81.8	81.0	81.3	84.2
Glu	0.9	92.3	54.5	68.6	74.1
Gln	0.3	100.0	47.6	66.7	77.4
GPC	0.2	61.5	78.3	72.2	67.4
Asc	0.8	63.6	90.9	81.8	75.4
Left Thalamus					
tNAA	1.3	61.5	90.5	79.4	82.1
tCho	0.3	84.6	61.9	70.6	72.5
mI	1.1	100.0	72.2	83.9	89.7
NAA	0.9	100.0	61.5	85.7	76.9
Right Putamen					
Glx	1.4	69.2	78.9	75.0	78.9
tCho	0.3	71.4	76.2	74.3	76.2
mI	1.2	72.7	88.2	82.1	77.5
Glu	1.1	76.9	84.2	81.3	82.6
GABA	0.3	63.6	84.6	75.0	77.6
Left Putamen					
Glx	1.6	83.3	72.2	76.5	84.1
Glu	1.3	83.3	76.9	78.9	78.2
GABA	0.46	100.0	66.7	78.6	86.7
PE	0.95	100.0	80.0	85.7	100.0

**Table 4:** Correlations between sleep variables and metabolite ratios.

		**Correlation Coefficients**	***p*****-values**
Left Thalamus			
SaO_2_ Baseline	mI	0.657	0.02
Right Putamen			
AHI	tCho	0.591	0.034
SaO_2_ Baseline	GABA	0.696	0.025
Left Putamen			
SaO_2_ Baseline	PE	0.969	0.031
SaO_2_ Nadir	NAA	0.955	0.011
	PE	−0.991	0.009

**Table 5:** Results are shown of the reproducibility study for the two subjects for three different 4D EP-JRESI measurement in the same day showing the co-efficient of variation (COV) in %.

**Metabolites →**	**tNAA**	**tCho**	**Glx**	**Glu**	**NAA**	**mI**
Healthy Controls						
Right Putamen	03.65	01.89	01.31	12.46	0.96	05.45
Left Putamen	05.06	07.65	12.21	09.85	13.44	05.88
OSAS						
Right Thalamus	02.79	13.51	03.85	10.16	07.29	06.04
Left Thalamus	02.48	05.86	06.25	15.29	14.69	17.13
